# A linear mixed model approach to gene expression-tumor aneuploidy association studies

**DOI:** 10.1038/s41598-019-48302-1

**Published:** 2019-08-16

**Authors:** Douglas W. Yao, Nikolas G. Balanis, Eleazar Eskin, Thomas G. Graeber

**Affiliations:** 1 Department of Molecular, Cell, and Developmental Biology, University of California, Los Angeles, CA USA; 2 Department of Molecular and Medical Pharmacology, David Geffen School of Medicine, University of California, Los Angeles, CA USA; 3 Crump Institute for Molecular Imaging, David Geffen School of Medicine, University of California, Los Angeles, CA USA; 4 Jonsson Comprehensive Cancer Center, David Geffen School of Medicine, University of California, Los Angeles, CA USA; 5 California NanoSystems Institute, David Geffen School of Medicine, University of California, Los Angeles, CA USA; 6 Department of Computer Science, University of California, Los Angeles, CA USA; 7 Department of Human Genetics, University of California, Los Angeles, CA USA

**Keywords:** Cancer genetics, Cancer genomics, Genome informatics

## Abstract

Aneuploidy, defined as abnormal chromosome number or somatic DNA copy number, is a characteristic of many aggressive tumors and is thought to drive tumorigenesis. Gene expression-aneuploidy association studies have previously been conducted to explore cellular mechanisms associated with aneuploidy. However, in an observational setting, gene expression is influenced by many factors that can act as confounders between gene expression and aneuploidy, leading to spurious correlations between the two variables. These factors include known confounders such as sample purity or batch effect, as well as gene co-regulation which induces correlations between the expression of causal genes and non-causal genes. We use a linear mixed-effects model (LMM) to account for confounding effects of tumor purity and gene co-regulation on gene expression-aneuploidy associations. When applied to patient tumor data across diverse tumor types, we observe that the LMM both accounts for the impact of purity on aneuploidy measurements and identifies a new association between histone gene expression and aneuploidy.

## Introduction

Genomic instability refers to an increase in the rate of mutations and chromosomal aberrations in aggressive tumors^1,2^. An observable consequence of genomic instability is aneuploidy, which broadly refers to abnormal chromosome number or somatic DNA copy number^3^. Genomic instability is thought to promote tumorigenesis by deregulating oncogenes and tumor suppressor genes and increasing the genetic diversity of tumors^4–8^. Previous work investigating the mechanisms underlying genomic instability has uncovered a link between genomic instability and aberrant DNA replication machinery, which can be caused by abnormal chromosome number^9,10^. However, the mechanisms that cause genomic instability and/or enable the cell to tolerate genomic instability remain to be fully characterized. Gene expression profiling of tumors via next-generation sequencing technologies such as RNA-seq provides insight into the cellular mechanisms associated with phenotypes such as tumor aneuploidy. Recently, large amounts of tumor sequencing data from projects such as the The Cancer Genome Atlas (TCGA) have allowed researchers to conduct expression-based studies that identify genes whose expression is significantly associated with aneuploidy^11–13^, with the premise that genes whose expression levels are most strongly correlated with aneuploidy across patient tumor samples are most likely to have a mechanistic relationship with aneuploidy. These studies use a simple linear regression (SLR) model with gene expression level as the predictor variable and an aneuploidy metric calculated from DNA copy number data as the response variable to perform association testing for each gene individually. These studies shown that among all genes, the expression of those involved in the cell cycle are most significantly positively associated with aneuploidy. Recent studies^12,13^ have also proposed that genes expressed by immune cells are negatively associated with aneuploidy and are evidence of a relationship between aneuploidy and immune evasion.

However, confounding factors such as sample purity that affect both gene expression and DNA copy number measurements in tumors can lead to spurious correlations between expression and measured aneuploidy. There is extensive evidence that tumor purity, if not properly accounted for, confounds common genome-wide expression-based analyses on tumor samples^14,15^. Moreover, gene co-regulation can also lead to spurious correlations. As a result of co-regulation, the expression of any gene that is in the same pathway or transcriptional program as a true causal gene will be associated with aneuploidy despite not having a causal effect on aneuploidy. In the context of association testing, gene expression co-regulation acts a confounder and will result in many false positive associations being identified, where we consider a true positive association to represent a gene with an experimentally verifiable functional relationship with aneuploidy. Gene Set Enrichment Analysis (GSEA)^16^ and related enrichment analysis tools^17,18^ can provide insight into functional groups of genes that are jointly associated with a phenotype, but these methods do not distinguish driver genes from passenger genes and thus by themselves cannot identify specific genes that have a direct mechanistic relationship with the phenotype. For example, GSEA has shown that the expression of almost all genes that encode proteins with catalytic or regulatory functions in the cell cycle—over 400 genes as defined by the Reactome Pathway Database—is highly significantly associated with aneuploidy in gene expression-aneuploidy association studies^12,13^. However, no clear distinction exists between associations in experimentally verified causal genes for aneuploidy^19–24^ and in the remainder of the significantly associated cell cycle genes, which vastly outnumber the verified causal genes and likely represent non-causal passenger genes.

In this study, we used a linear mixed-effects model (LMM) to perform association testing of gene expression vs. aneuploidy while accounting for confounding effects due to purity and gene co-regulation. The LMM was originally proposed as a method to correct for confounding due to population stratification in genome-wide association studies (GWAS)^25–27^, and our application of the LMM to our study is motivated by conceptual similarities between GWAS and expression-aneuploidy association studies (See Discussion for more information). We applied the LMM to perform association testing between gene expression and aneuploidy in 22 tumor datasets from TCGA and METABRIC, observing that the LMM accounted for associations between gene expression and aneuploidy occurring due to purity differences between samples. The LMM also identified a novel association between the expression of histone genes and aneuploidy. Our results demonstrate the power of the LMM to correct for confounding and identify biologically interesting associations in settings outside of GWAS.

## Results

### Overview of the LMM

Here, we describe the motivation of the LMM and how to interpret results produced by the method. When performing association testing of expression vs. aneuploidy, if we knew a priori all possible confounders between gene expression and aneuploidy and had a way to measure them, we could include these confounders as fixed-effects covariates in the association testing model in order to account for their confounding effects. However, in reality most confounders between gene expression and aneuploidy are unknown or unmeasurable. Instead of including these confounders as fixed effects in the model, we can include a single variance component that will capture the correlation between expression across all genes for all pairs of samples. The rationale behind doing this is as follows: if there exists a confounder that affects the expression of many genes, then a pair of samples that have similar values of the confounder will have overall more similar expression profiles. Thus, we can view the overall similarity between the expression profiles across individuals as a proxy to the effects of all confounders on these individuals. When we perform association testing between gene expression and aneuploidy with this variance component in the model, we will identify associations that are not *fully explained* by the total correlation in expression profiles between samples.

The main drawback of the LMM approach is that overall similarity between expression profiles of individuals can reflect true biological activity in addition to confounding. For example, if the expression of a transcription factor is causal for aneuploidy but is also correlated with the expression of many other genes, in the variance component of the LMM the effects of the transcription factor on many genes will be indistinguishable from effects of confounding. Thus, because the association between the transcription factor’s expression and aneuploidy will be fully explained by the correlation in expression profiles between individuals, the transcription factor will not be identified by the LMM as a significant association. Although the LMM cannot identify these types of associations, the types of associations that *are* identified by the LMM likely have a more direct relationship with aneuploidy that is not induced through confounding or co-regulation with causal genes.

For *N* samples, under a simple linear regression (SLR) model (which is the model used by previous gene expression-aneuploidy association studies), the relationship between aneuploidy and gene expression for gene *k* is modelled as follows:

$${\boldsymbol{y}}={\beta }_{0}1+{\beta }_{1}{{\boldsymbol{x}}}_{k}+\varepsilon $$

where ***y*** is an *N*-vector of aneuploidy values, ***x***_*k*_ is an *N*-vector of expression values for a given gene *k*, *β*_0_ is the intercept, *β*_1_ is the slope coefficient, and ***ε*** is an *N*-vector of unmodelled effects. The strength of association between the *x*_*k*_ and *y* can be quantified by performing hypothesis testing on the ordinary least squares estimate of *β*_1_ to obtain a p-value against the null hypothesis that *β*_1_ = 0.

The LMM introduces a variance component ***u*** into the SLR model:

$${\boldsymbol{y}}={\beta }_{0}1+{\beta }_{1}{{\boldsymbol{x}}}_{k}+{\boldsymbol{u}}+\varepsilon $$

We define ***u*** as a multivariate normal random variable with mean **0** and covariance ${\sigma }_{g}^{2}{\boldsymbol{K}}$. We define ***K*** as the sample covariance matrix of the sample-by-gene expression matrix, where all expression values are scaled and centered to equal mean and variance per gene. Conceptually speaking, ***u*** explains some fraction of variance in the phenotype *y* that is influenced by the correlation structure in expression profiles between samples. For example, if *x*_*k*_ is correlated with the expression of many other genes, changes in *x*_*k*_ will also involve changes in the expression of all correlated genes, which will cause samples with different values of *x*_*k*_ to be less correlated overall as captured by ***u***. As a result, a large proportion of variance in *y* will be explained by *u* rather than *x*_*k*_, which will reflect in a less significant p-value when we perform hypothesis testing on the generalized least squares (GLS) estimate of *β*_1_. On the other hand, if *x*_*k*_ is *not* correlated with the expression of other genes, then *x*_*k*_ will vary independently of the correlation structure of the samples, which will allow us to more confidently attribute changes in *y* to changes in *x*_*k*_ rather than *u* (See Methods). Thus, genes whose expression is relatively uncorrelated with the expression of other genes, but still correlated with aneuploidy, will result in the most significant associations in the LMM.

### LMM corrects for confounding due to purity

The degree of aneuploidy of a tumor sample can be summarized by quantifying the total number of DNA copy number changes across the genome. As an alternative to the copy number/aneuploidy scores used by Davoli *et al*. and Taylor *et al*., we defined a copy number metric called the integrated CNA (ICNA) score^28^, which can be easily calculated from segmented relative DNA copy number data (Methods) without the application of external algorithms unlike the other metrics. We calculated the ICNA scores of 7,802 samples across 21 tumor types from TCGA and 1,895 breast cancer samples from METABRIC^29^ (Supplementary Table S1). We first performed association testing of gene expression vs. ICNA score under a simple linear regression model, which is the approach taken by previous expression-aneuploidy association studies. After applying GSEA to our association testing results, we observed positive enrichment of cell cycle gene sets and negative enrichment of immune gene sets, which is consistent with results from previous studies^12,13^ (See Supplementary Table S2a for association testing p-values and Supplementary Table S2b for GSEA results).

**Figure 1:**
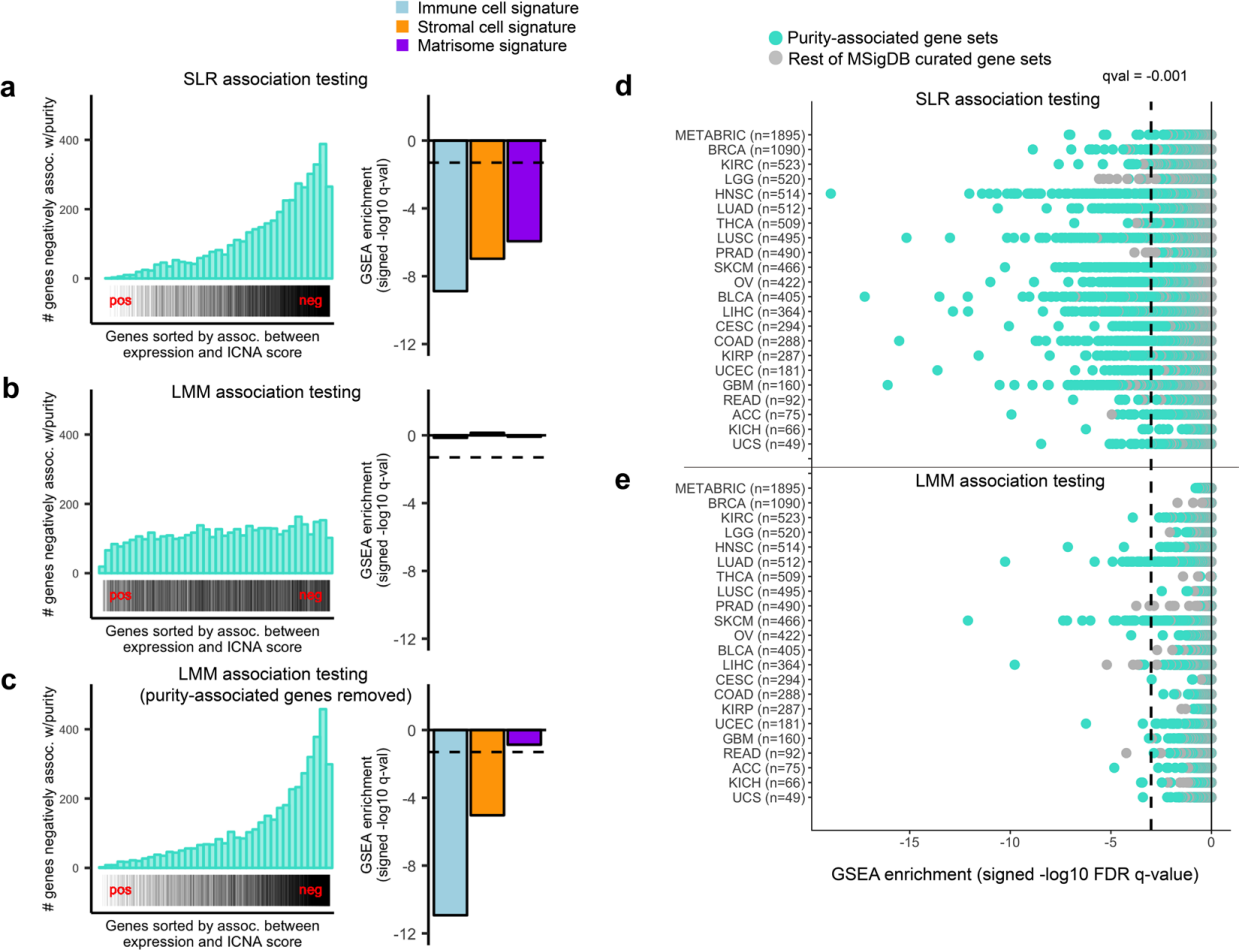
LMM accounts for confounding effects of purity on gene expression-ICNA score associations. (**a**–**c**) Association testing of expression vs. ICNA score was performed using three models: SLR, LMM, and LMM with all purity-associated genes removed from the intersample correlation matrix. Results are shown for the largest TCGA dataset (n = 1,090) consisting of breast invasive carcinoma (BRCA) samples. **Left** Rug plot and histogram. x-axis: List of all genes sorted by association strength—i.e. correlation coefficient—between their expression and ICNA score, with most positively correlated genes toward the left and most negatively correlated genes toward the right. Purity-associated genes are indicated in black. y-axis: Histogram of purity-associated genes that fall into each bin. **Right** Bar plot showing the GSEA enrichment of immune cell, stromal cell, and matrisome signatures in the association testing results of expression vs. ICNA score. Dotted line indicates an FDR q-value of 0.05. (**d**,**e**) Negatively enriched gene sets in the SLR and LMM association testing results for expression vs. ICNA score for 21 TCGA tumor types and METABRIC (breast cancer).

In order to examine the impact of purity on our association testing results, we obtained purity estimates for all tumors from Aran *et al*.^14^, who leveraged gene expression, DNA copy number, DNA methylation, and histological image data to define a consensus purity estimate (CPE) for each sample. We defined a set of purity-associated genes as genes whose expression was significantly correlated with CPE with Bonferroni-corrected p-value less than 0.01. We also defined a set of purity-associated gene sets that were significantly enriched as measured by GSEA with false discovery rate less than 0.25. We observed substantial overlap between purity-associated and ICNA score-associated genes (Fig. 1a), suggesting that purity had a confounding effect on the associations between gene expression and ICNA score. We observed the same trend when comparing purity-associated gene sets to ICNA score-associated gene sets (Fig. 1d).

We then applied the LMM to perform association testing of expression vs. ICNA score (See Supplementary Table S3a,b for association testing p-values and GSEA results) and observed that the overlap between purity-associated and ICNA score-associated genes/gene sets was mostly removed, supporting that the LMM accounted for the confounding effects of purity (Fig. 1b,e).

To verify that the LMM was specifically correcting for confounding due to tumor purity rather than simply introducing noise, we removed all purity-associated genes (p-value < 0.01 Bonferroni correction) prior to construction of the gene correlation matrix and performed association testing using the LMM. In this case, the LMM variance component contained essentially no purity information. In the LMM output, we observed that the purity-associated genes now again overlapped with the ICNA score-associated genes (Fig. 1c), confirming that the impact of purity on ICNA score was specifically captured and accounted for by the full variance component in the LMM.

### Histone gene expression directly associated with aneuploidy

**Figure 2:**
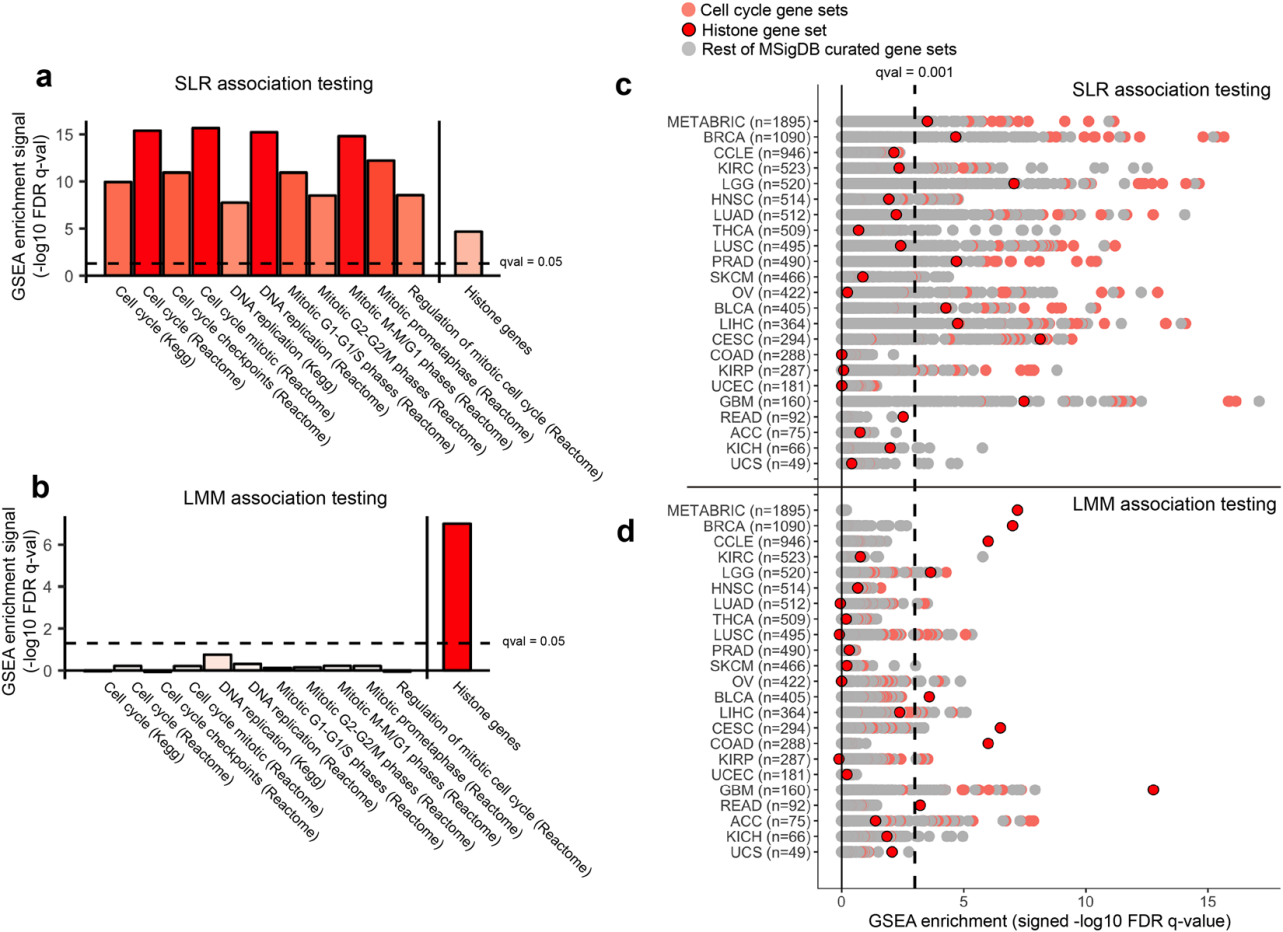
LMM enriches for associations between histone gene expression and ICNA score. (**a**,**b**) Bar plots showing the GSEA enrichment strengths of cell cycle gene sets and the histone gene set in the SLR and LMM association testing results of gene expression vs. ICNA score. Results are shown for TCGA BRCA samples only. (**c**,**d**) All positively enriched gene sets in the SLR and LMM association testing results for expression vs. ICNA score for 21 TCGA tumor types, METABRIC (breast cancer), and CCLE (cancer cell lines). Cell cycle gene sets are the same ones used in (**a**,**b**).

In the LMM association testing results, we observed that many of the strongest associations consisted of histone genes. To determine the overall enrichment of all histone genes within the association testing results, we defined a histone gene set to include all genes that encode the core histone proteins and histone H1 (See Supplementary Table S4 for full list of genes). In the SLR association testing results of gene expression vs. ICNA score, we observed that the enrichment of the histone gene set was present but overshadowed by the enrichment of cell cycle gene sets (Fig. 2a,c). On the other hand, in the LMM association testing results, the enrichment of all cell cycle gene sets disappeared, while the enrichment of the histone gene set remained highly significant (Fig. 2b,d). Even though the histone gene set is a small subset of the cell cycle gene sets, the fact that the histone gene remains significant, but not the cell cycle gene set as whole, means that the non-histone genes in the cell cycle gene set are not enriched, diluting the histone signal and causing the overall enrichment of the cell cycle gene set to be non-significant. This supports that histone gene expression is associated with ICNA score independently of co-regulation with genes within the cell cycle.

We also repeated this analysis using copy number scores defined by Davoli *et al*. and and Taylor *et al*., observing that the enrichment of the histone gene set in the LMM association testing results was overall weaker but still significant for several tumor types (Supplementary Fig. S1).

### Large sample sizes required for LMM to correct for confounding effects

When comparing the association testing results from LMM to the results from SLR for each dataset, we observed that the degree of similarity in the results from these two different methods was negatively correlated with the amount of samples in each dataset, supporting that the LMM was able to separate out direct associations best when given information from more samples (Supplementary Fig. S2a). For many TCGA tumor types other than BRCA, we observed an attenuation but not a complete removal of the enrichment of both cell cycle and purity-related gene sets, which may be explained by their small sample sizes (Figs 1e and 2d). As confirmation, we took random subsets of varying sizes from the BRCA dataset and applied the LMM to these subsets, observing that the enrichment strength of cell cycle and purity-related gene sets was negatively correlated with the amount of samples in the subsets, and that the enrichment was only completely non-significant at large sample sizes around 800 (Supplementary Fig. S2b,c).

## Discussion

In this study, we demonstrate that the LMM can correct for confounding due to purity in a gene expression-aneuploidy association study and identifies a new association between histone gene expression and aneuploidy. The LMM does not require any external information other than gene expression measurements to correct for confounding due to purity, relying on the fact that purity influences the expression of many genes, and thus the expression of all purity-affected genes will be correlated with each other across samples. The LMM also potentially corrects for other unknown confounders such as batch effect, which are also known to influence the expression of many genes^30–32^.

We also report an association between histone gene expression and aneuploidy. The fact that this association is significant under the LMM supports that the association does not occur due to histone genes simply being part of a large pathway that is upregulated as a whole in more aneuploid cells. This an unexpected result given that histone genes are known to only be transcribed during DNA replication and are coupled with the S phase of the cell cycle^33^. The fact that histone gene expression is significantly associated with aneuploidy under the LMM, whereas the expression of other genes involved in DNA replication and the S phase of the cell cycle is not significantly associated, suggests that histone gene expression occurs independently of the cell cycle in aneuploid tumors, possibly to accommodate the increased DNA content arising from higher ploidy^13^.

One of the main drawbacks of the LMM association testing approach is that many drivers of cancer phenotypes are transcription factors or other genes within gene pathways^34–37^, whose expression by nature may be associated with that of many other genes and as a result may not be significantly associated with the phenotype under the LMM. However, we propose the LMM model here as a discovery tool. We can think of the LMM as having a smaller false positive rate than simple linear regression, since genes that are correlated with many other genes can be falsely correlated with aneuploidy, and these genes are being account for by the LMM. Our finding that histone gene expression is associated with aneuploidy is not reported in any previous expression-aneuploidy association studies and thus represents a new result enabled by the LMM approach. Both simple linear regression results and LMM results are discovery tools, each with a different set of strengths and caveats to keep in mind during interpretation. Ultimately, experimental follow-up is required.

In the context of association studies, the LMM approach was originally developed as a tool to correct for confounding due to population stratification in GWAS^25–27^. GWAS and expression-aneuploidy association studies are conceptually very similar. In a GWAS, the association between the minor allele count of each SNP and a phenotype is computed. In an expression association study, the association between the expression levels of each gene and a phenotype (in our case, aneuploidy) is computed. Both types of studies aim to identify significantly associated SNPs or genes respectively in order to elucidate functional mechanisms of the phenotype.

In a GWAS, there is a phenomenon known as population stratification that leads to unwanted correlations between SNPs, which in turn results in spurious associations between these SNPs and the phenotype. This phenomenon is analogous to the presence of gene pathways or confounders such as tumor purity in expression association studies, which lead to correlations between genes. This in turn may result in spurious associations between these genes and the phenotype. Thus, our motivation for using the LMM comes from observing this similarity between GWASes and expression association studies, and our implementation of the LMM in our study is identical to how it would be implemented in a GWAS, with the only difference that we are looking at gene expression levels rather than SNP minor allele counts.

## Methods

### Data sets

RNA-sequencing data for tumor samples across 21 TCGA tumor types was processed using the TOIL pipeline^38^. We downloaded the log transformed RSEM expression counts directly from UCSC Xena. We downloaded segmented SNP-array based copy number data for 21 TCGA tumor types from the NCI Genomic Data Commons. We obtained the Davoli and Taylor scores of TCGA samples as part of the Supplementary Data of their respective papers^12,13^. We obtained purity information for TCGA samples across 21 tumor types from the Supplementary Data of Aran *et al*.^14^. The paper uses three independent algorithms that leverage gene expression, methylation, and DNA copy number information respectively to quantify the proportion of cancer cells in each TCGA sample, as well as an immunohistochemistry analysis that estimates purity by image analysis of haematoxylin and eosin stain slides. The paper defines a consensus purity value for each TCGA sample based on the median of the results from the four methods, which we used as the purity value in our analyses. We obtained processed microarray expression data and copy number data for CCLE samples from the Broad Institute’s CCLE data portal. We obtained processed microarray expression data and copy number data for METABRIC samples from cBioPortal via the cgdsr R package. We restricted all our analyses to include HUGO coding genes only.

### Integrated copy number alteration (ICNA) score calculation

Starting from the processed DNA copy number data in.seg file format, we calculated the ICNA score of each sample as the sum of lengths of each segment of copy number change weighted by the relative copy number change of the segment. For sample *n*, let *L*_*in*_ and *C*_*in*_ be the length and relative copy number change for a particular segment *i*.

$$ICNA\,score(n)=\sum _{i\in all\,segment{s}_{n}}\,({L}_{in}\times {C}_{in})$$

### Association testing using simple linear regression

To carry out association testing of each gene’s expression vs. aneuploidy under SLR, we used the following model:

$$y={\beta }_{0}+{\beta }_{1}{x}_{k}+\varepsilon $$

*y* is ICNA score, *x*_*k*_ is the log-transformed and normalized expression of gene *k*, *β*_0_ is a constant that represents the intercept, and *ε* is a normally distributed error term. We measured the strength of association between *x*_*k*_ and *y* by performing a t-test on the ordinary least squares (OLS) estimate of *β*_1_ and obtaining a p-value against the null hypothesis *β*_1_ = 0. This was done using the lm function in R.

### Association testing using the linear mixed model

The LMM introduces a random effect ***u*** into the simple linear model. We represent the model in vector notation:

$${\boldsymbol{y}}={\beta }_{0}1+{\beta }_{1}{{\boldsymbol{x}}}_{k}+{\boldsymbol{u}}+\varepsilon $$

where ${\boldsymbol{u}} \sim {\mathscr{N}}(0,{\sigma }_{g}^{2}{\boldsymbol{K}})$ and $\varepsilon  \sim {\mathscr{N}}(0,{\sigma }_{e}^{2}{\boldsymbol{I}})$. ***K*** is an intersample covariance matrix that was calculated from the sample expression data as follows. Given an *m* × *n* expression matrix with *m* samples and *n* genes, for each gene, we standardized the expression values to mean 0 and variance 1 by subtracting the mean and dividing by the standard deviation of that gene’s expression values. If we let ***Z*** be the *m* × *n* standardized expression matrix, we defined ***K*** as the *m* × *m* covariance matrix of ***Z***, which we calculated as $\frac{{\boldsymbol{Z}}{{\boldsymbol{Z}}}^{T}}{n}$.

Let ${\boldsymbol{V}}={\sigma }_{g}^{2}{\boldsymbol{K}}+{\sigma }_{e}^{2}{\boldsymbol{I}}$. Under the LMM, the joint distribution of the aneuploidy values ***y*** follows a multivariate normal distribution with mean ${\beta }_{0}1+{\beta }_{1}{{\boldsymbol{x}}}_{k}$ and covariance ***V***:

$${\boldsymbol{y}} \sim {\mathscr{N}}({\beta }_{0}1+{\beta }_{1}{{\boldsymbol{x}}}_{k},{\boldsymbol{V}})$$

${\sigma }_{g}^{2}$ and ${\sigma }_{e}^{2}$ are both scalars that were optimized from our sample data by maximizing the log-likelihood function of the multivariate normal model:

$${\hat{\sigma }}_{g}^{2},{\hat{\sigma }}_{e}^{2}={{\rm{argmax}}}_{{\sigma }_{g}^{2},{\sigma }_{e}^{2}}-\frac{1}{2}[n\,\mathrm{log}(2\pi )+\,\mathrm{log}\,|{\boldsymbol{V}}|+{({\boldsymbol{y}}-{\beta }_{0}1)}^{T}{{\boldsymbol{V}}}^{-1}({\boldsymbol{y}}-{\beta }_{0}1)]$$

Once we obtained our estimates of ${\sigma }_{g}^{2}$ and ${\sigma }_{e}^{2}$, we performed hypothesis testing on the generalized least squares (GLS) estimate of *β*_1_. The GLS estimator for all model coefficients, represented in vector notation as $\hat{\beta }$, is given by:

$$\hat{\beta }={({{\boldsymbol{X}}}^{T}{{\boldsymbol{V}}}^{-1}{\boldsymbol{X}})}^{-1}{{\boldsymbol{X}}}^{T}{{\boldsymbol{V}}}^{-1}{\boldsymbol{y}}$$

where ***X*** is the design matrix of the model. $\hat{\beta }$ is the best linear unbiased estimator (BLUE) for the coefficients in a linear model where the residuals have a known covariance structure, which is given by ***V*** in our case. $\hat{\beta }$ is asymptotically distributed according to a multivariate normal distribution with known mean and variance:

$$\hat{\beta } \sim {\mathscr{N}}\,(\beta ,\frac{{({{\boldsymbol{X}}}^{T}{{\boldsymbol{V}}}^{-1}{\boldsymbol{X}})}^{-1}}{n})$$

where *n* is the number of samples. We performed hypothesis testing on the GLS estimate of *β*_1_, which follows a univariate normal distribution, to obtain a p-value against the null hypothesis that *β*_1_ = 0, which represents the strength of association between *x*_*k*_ and *y* with the intersample correlation structure taken into account. We performed all LMM parameter optimization and hypothesis testing using the Python module PyLMM (https://github.com/nickFurlotte/pylmm).

### Gene set enrichment analysis

#### Gene set information

We conducted gene set enrichment analysis on our association testing results using the command line pre-ranked GSEA application downloaded from the Broad Institute’s website. We ran GSEA using all 1,329 canonical pathway gene sets (C2 CP) from the Broad Institute’s online Molecular Signatures Database (MSigDB) with four additional gene sets: an immune cell gene set and a stromal cell gene set defined in a study by Yoshihara *et al*. on tumor purity^39^, a histone gene set consisting of all genes that code for histone proteins, and a ribosomal protein gene set consisting of all RPS and RPL genes. We removed all histone genes from the canonical pathway gene sets when running GSEA, since some gene sets—namely Chromosome Maintenance (Reactome), Telomere Maintenance (Reactome), and Packaging of Telomere Ends (Reactome)—contained many histone genes that caused them to be significantly enriched in the LMM association testing results. Upon removal of all histone genes from these gene sets, we observed that the enrichment of the remaining genes—which were comparable in number to the histone genes removed—was no longer significant, demonstrating that the enrichment of the original gene sets was due to only the histone genes.

#### FDR q-value extrapolation

GSEA uses a permutation test to calculate the p-values and false discovery rates (FDR) of every gene set’s enrichment. In our results, we observed that very highly enriched gene sets had an FDR q-value of 0 even after running >10 million total permutations (10,000 permutations per gene set × 1,333 gene sets). In order to compare the relative enrichments of these highly enriched gene sets, we used LOESS to extrapolate their q-values from the normalized enrichment score (NES) vs. −log10 q-value plot for all gene sets with nonzero q-values within each dataset. This was done using the loess function in R.

## Supplementary information

Supplementary Figures

Supplementary Table 1

Supplementary Table 2

Supplementary Table 3

Supplementary Table 4

## Data Availability

Log-transformed RSEM gene expression counts for all TCGA tumor samples is available from UCSC Xena, https://xenabrowser.net. Segmented DNA copy number data for all TCGA tumor samples is available from the NCI Genomic Data Commons, https://portal.gdc.cancer.gov. Gene expression counts and segmented DNA copy number data for all CCLE samples is available from the CCLE portal, https://portals.broadinstitute.org/ccle/data. Gene expression counts and segmented DNA copy number data for METABRIC samples is available from cBioPortal, http://www.cbioportal.org.
