# Supplementary material for: A linear mixed model approach to gene expression-tumor aneuploidy association studies
Source: Sci Rep. 2019 Aug 16;9:11944. doi: 10.1038/s41598-019-48302-1 (PMC6697733; doi:10.1038/s41598-019-48302-1)
Supplement: Supplementary file 1 — Supplementary Figures [file 41598_2019_48302_MOESM1_ESM.pdf]

Supplementary Figures for "A linear mixed model approach to gene expression-tumor aneuploidy association studies"

Douglas W. Yao, Nikolas G. Balanis, Eleazar Eskin, Thomas G. Graeber

Supplementary Figures 1-2

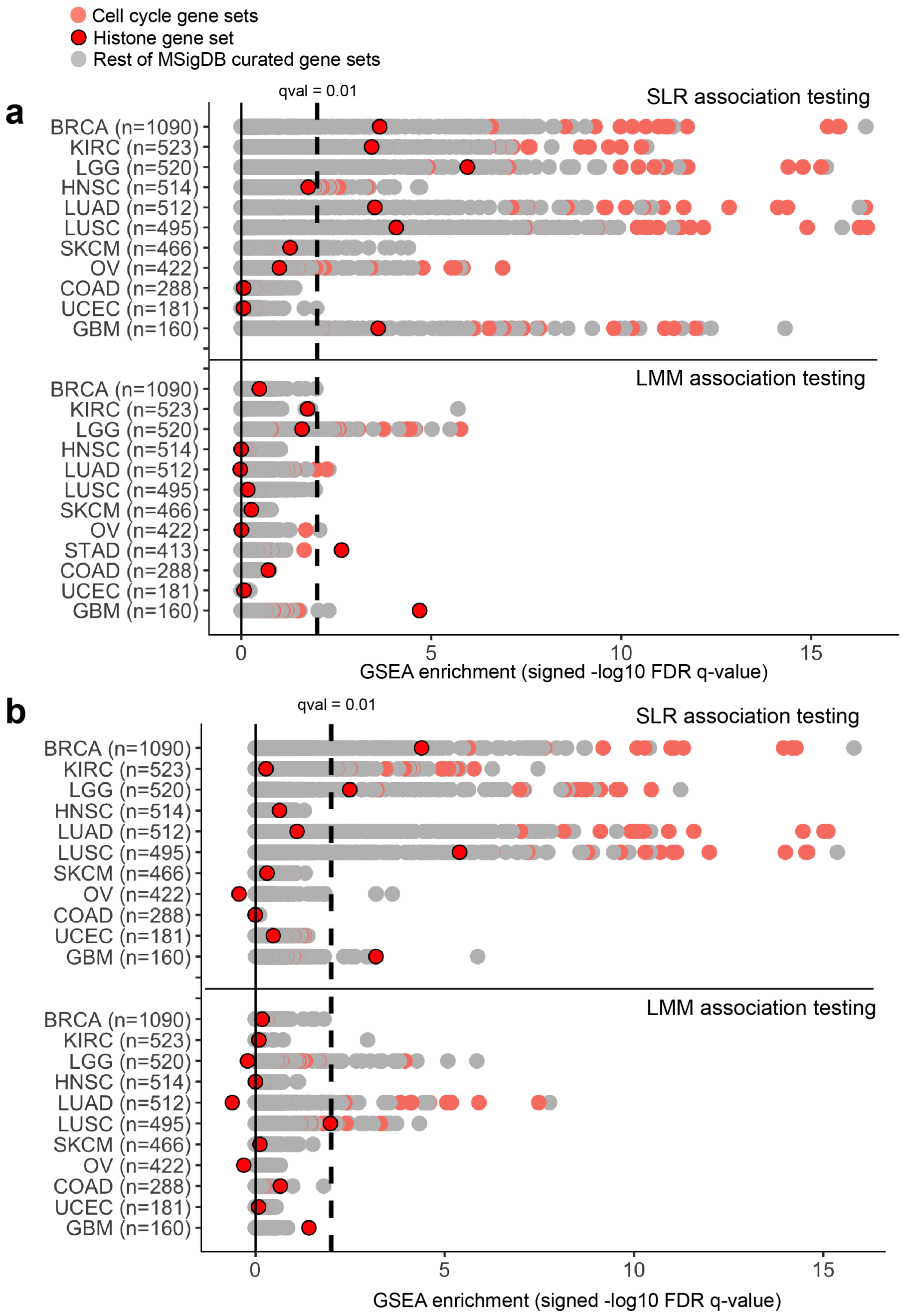

Figure S1: (**a-b**) Same as Fig. 2b-c, but showing association testing results using Davoli scores (**a**) and Taylor scores (**b**) rather than ICNA scores.

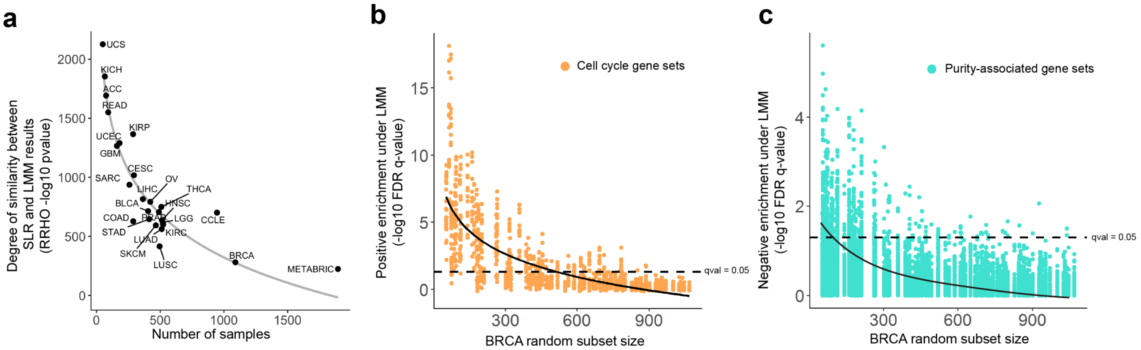

Figure S2: **LMM association testing results depend on sample size.** **a** Scatterplot of similarity between SLR and LMM association testing results for each dataset vs. the sample size of the dataset. Rank-rank hypergeometric overlap (RRHO) was used to quantify the degree of similarity between the lists of genes ranked by their association p-values under SLR and LMM association testing. **b, c**. Random subsets of varying size were taken from the TCGA BRCA dataset, and the enrichment levels of all cell cycle gene sets and purity-associated gene sets from LMM association testing of expression vs. aneuploidy are plotted against subset size.
